# Illustrations of the Origin and Propagation of Certain Epidemic Disorders

**Published:** 1859-01

**Authors:** T. Herbert Barker


### ILLUSTRATIONS OF THE ORIGIN AND PROPAGATION OF CERTAIN EPIDEMIC DISEASES.

By T. HERBERT BARKER, M.D., F.R.C.S.

Although hygienic medicine and epidemiology may be considered as sciences still in their infancy, sufficient matter-of-fact has been instilled into them to render common-place (and certainly common-place here) any description of the intimate relationship which exists between moral degradation (in which I include all uncleanliness) as one fact, and disease as another fact consequent on the first. But while in the mere admission of this general truth an every day thought is expressed, it were impossible for the epidemiologist to allow such expressions to stand for the *all* of his labour. It is his business, as a practical worker in his vocation, to take up the particulars of the general fact, and fixing on particular maladies which in their unity give rise to the term disease, to trace out of the *general* producing causes those *individual* causes which light up individual disorders.

In this direction, indeed, epidemiologists are working with great labour; and in what I shall read to-night, I shall offer nothing truly novel of my own. In fact, to the labours of many co-workers with me and for me, I am indebted for the materials out of which my paper is constructed.

There is, nevertheless, one special object in this communication, that, namely, of attempting to give some exactness to the study of epidemic diseases.

I wish to keep prominently before the Society the following points :—

1. Given a certain class of disorders called epidemic and endemic, how many and which of these have their origin in poisons extant only in the external world, and not reproducible in, nor transmissible from the affected body ?

2. How many and which of the epidemic and endemic disorders have their origin in poisons which are reproducible in the body, and transmissible from one body to another?

3. How many and which of the epidemic and endemic diseases have their origin not in any specific poison, but in atmospheric peculiarities affecting all?

Some late inquiries into the mode of propagation of epidemics have fixed my mind so much to the study of these questions, that it seems to me now as if the first step in systematic epidemiology were centered in them. This remark, I know, is open to discussion, and it will not fail in its object if it calls forth discussion.

Further, I would even suggest the breaking down of the old and somewhat puerile distinction of epidemic and endemic disease, and the setting up of a new classification of epidemics and endemics into three divisions, according as they have their origin in one or other of the three causes given above. I shall so far trespass on custom, at all events on the present occasion, as to base all my observations relating to the propagation of epidemic disease on this classification.

Class I. Diseases not transmissible—originating in a poison not reproducible in the body.

Class II. Diseases transmissible—originating in a poison which is reproducible in the body.

Class III. Diseases not transmissible—originating in meteorological variations.

To the first of the classes of diseases named above, four diseases of the diffusive type pertain in this country. These are, ague, diarrhœa, remittent fever, a peculiar kind of noncontagious fever, having in most cases the qualities of typhoid fever, which Dr. Murchison proposes to call pythogenic (πύθομαι, putresco, γεννάω, I produce) fever, to point at its origin from putrid animal effluvia; and which from being confined almost exclusively to the vicinities of cesspools, foul drains, sewers, or receptacles for decomposing organic matter, has received the various names of cesspool fever, nightsoil fever, and the like.

I shall suffer ague to pass unnoticed on this occasion, in order to dwell at greater length on the special pythogenic fever which has been referred to, and on the diarrhœal disorder.

That there is a fever not transmissible, which owes its origin to cesspool emanations, is a view held by many authorities. This view is strengthened by certain experiments of my own, recorded in the Fothergillian Essay on *Malaria*, and published lately in the *Sanitary Review*, from which it is shown that a peculiar and analogous feverish disorder can be produced in inferior animals, by the inhalation of cesspool air.

In these experiments not only was the production of a special disease indicated, but two other facts, [illegible] that removal after exposure to the poison is not productive of immediate restoration to health, and that the removal of an animal diseased from the poison, and the placing of it with a healthy animal, does not propagate the disorder.

Comparing these experimental facts with what I have observed in the human subject, the two series of observations tally in a remarkable manner. One of the common forms of fever which we have in Bedford, is of the class of which I now speak; but, as it does not often occur in isolated cases, and as it cannot often be traced direct to its cause, much difficulty exists in isolating it completely. After issuing some inquiries to medical friends last year, I obtained, however, many well marked illustrations of the local fever which I am now considering. Some of the cases were so clearly traced to their origin, that the course of the disease reads like an improvised experiment on the human subject.

The following illustration, occurring in the person of a well-known member of our profession, Dr. Swayne, of Bristol, and reported to me by himself, is perhaps, one of the best authenticated cases on record. I, therefore, give it entire.

"In the night of November 27th, 1852, I was called up to attend a labour in a neighbouring street. I arrived at the house about two in the morning. As I entered, I met some nightmen, who were engaged in emptying a cesspool connected with the drains in the house, which had not been disturbed for thirty years. The stench was most intolerable. I immediately experienced a feeling of nausea and headache, attended with some prostration of strength. However, I attended my patient upstairs in her confinement. All the doors communicating with the lower part of the house had been kept closely shut, and chlorine had been used freely in the staircase. The result was that no harm came to my patient, who recovered well. It was not so with myself. From that time I began to lose my appetite, to sleep badly, and be feverish at night, and also to suffer occasionally from headache. This went on until December 15th, 1852, when I was attacked with typhus. At first I suffered very much from headache, which was sometimes very intense. There were no gastro-enteric symptoms. The headache was much relieved by leeching and cold lotions, and subsided after a few days. The fever, however, continued without much abatement for nearly a month, when I began to mend, and in less than six weeks was able to go out a little. The quinine treatment of fever was at that time much in vogue, and was tried in my case. It did not, however, appear to cut short the fever in any way, although my medical attendants considered that it rendered my subsequent convalescence more speedy."

Another illustration, very striking from its isolation from sources of fallacy, is supplied me by my friend Mr. W. R. Milner, of Wakefield.

"Some years ago," writes Mr. Milner, "I attended a succession of fever cases in a row of cottages built quite on the top of a conical hill, so that the ground fell from them in every direction, and there were no buildings within several hundred yards of them. The cottages either belonged to, or were in the occupation of, the overseers of the poor of that township, and were used as residences for pauper families. I learned that they were scarcely ever free from fever. The inhabitants assured me that there were no collections of filth or other nuisances about them. One day, when I was riding at some distance from them, and looking towards the back of the cottages, my attention was arrested by a broad dark line running down the side of the hill. I found on examination that this arose from the coarse rank grass growing on the side of a channel, down which ran the overflowings of a long shallow pond, close to the backs of the cottages, and overlooked by the back windows. The pond was filled with the accumulated filth of years, and was so covered with a mass of green confervæ, oscillatoriæ, etc., that it was easily overlooked, and it was not at all visible from the road leading to the cottages. The evil arising from it being pointed out to the overseers, the pond was cleared out and filled up. I remained between two and three years in that neighbourhood; and, during that time, no fresh cases of fever occurred in these cottages."

I have received from various friends many further illustrations of the origin of a specific disease of the kind now being considered. I could not introduce these in full, but the following illustrations of an endemic typhus, the source of which, and the removal, are alike remarkable, must not be left unnoticed.

The village of Cople, near Bedford, had long been notorious as the scene of a constant endemic fever, and the register shows how considerable was the mortality from this cause. The village is low-lying; and by the side of the street, along a considerable part of the village, was a large wide open ditch, which, in rainy seasons, conveyed the water from the neighbouring hills to the brooks; in fact, in wet seasons, it was a regular water-course. In dry seasons it was partly filled with stagnant water in many places; in other places it was merely covered with mud and decomposing vegetable matters, and was also the receptacle for privy-matters, and every kind of filth. It was considered that this ditch had something to do with the frequency of fever in the village, and on representation being made to the proprietor of the parish, the Duke of Bedford, a deep water-course was made through the fields, at a distance from the cottages. The old shallow ditch was filled up, and converted into garden-ground for the cottagers. Not a single case of fever has since been known in this village. The change has been effected eight years, and is so marked as to have excited the notice of every inhabitant. The water supply remains in the same condition as it did in the fever periods.*

Thus by two series of inquiries, one derived from experiment, the other from experience, the evidence of a *fever-producing emanation* from the cesspool is well defined. It were a matter of great practical value to ascertain, what is the nature of the producing poison?

If I am not mistaken, there are poisons emanating from the cesspool having different properties. There is a poison which produces fever, and from the fact that this fever is, in

---

* The reader will find similar illustrations of the origin of fever in an excellent pamphlet, recently published at Oxford, by Professor Acland, entitled "Fever in Agricultural Districts". The fever village therein graphically depicted, is but a fair specimen of very many others to be found in our agricultural districts. I should add that the sanitary condition of Cople and the neighbouring village Willington, has been considerably improved by the erection, by the Duke of Bedford, of a great number of very superior cottages for the labouring classes. So excellent are these cottages in every arrangement, that they may justly be called "model cottages"; and our landed proprietors could not do better than copy their design.

its general form, the same, I opine that this poison is *one;* there is a poison which produces diarrhœa, and this I also presume to be one, and specific. I would consider briefly the nature of the fever-poison.

In my experiments on the lower animals, I endeavoured to ascertain, first, the nature of the gases which emanate from cesspools; and, secondly, the physiological effects of such individual substances as are to be found by analysis of the cesspool-air. I found that the emanations ordinarily evolved are sulphuretted hydrogen, carbonic acid, some alkaline body exceedingly volatile (*quasi* ammonia), and organic matter.* I found farther, on submitting animals to the action of these substances, separately, that certain peculiar symptoms were manifested which are certainly not without interest when compared with the symptoms induced by the compound emanation. I found that sulphuretted hydrogen breathed in proportions exceedingly small destroyed life, but not with the symptoms or pathology common to the cesspool fever. I found that a volatile alkaline body, and especially the compound of sulphuretted hydrogen and ammonia, did, on the other hand, when persistently exhibited, produce the fever, both in symptoms and pathology. Hence the inference on my mind is that, in cases of direct poisoning from cesspool air, the symptoms when they assume, as they ordinarily do, the typhoid character, are due to the direct inhalation of an alkaline poison. Dr. Richardson has lately illustrated this same point in a paper read before the Society; his mode of proceeding, and his inquiries altogether, have, it is true, a different origin and intention from mine, but the results are in the main the same.

Several excellent illustrations of the origin and propagation of remittent fever have been supplied me by different friends. This form of fever is rare in my neighbourhood, but I know it as distinct from the preceding types. From the illustrations in hand two are most striking, one by Dr. Spencer Thomson, of Burton-upon-Trent, the other by Mr. Watkins, of Towcester.

Dr. Thomson's case is as follows:—"A lady, who had been on a visit to a country town, returned home suffering from all the symptoms of remittent fever, the attack lasting severely for eight days. The only assignable cause for the attack, and this cause was sufficiently well marked, was, that

* Dr. Odling, who has made similar inquiries, and Dr. Letheby also, have observed the same facts; and Dr. Odling has opined that the alkaline emanation is a peculiar form of ammonia called ethylamine.

while she was on her visit, a stagnant pool situated close to the house in which she resided, was in the process of being emptied and cleansed; the mud and decomposing matter being thrown out on to the banks of the pool. The pool had long been the receptacle for a part at least of the town sewage. The decomposing refuse thus thrown out of the pool was left exposed to the heat of the sun, and to the inhalation of the emanations arising from this source my patient persistingly traced the origin of her malady, which quickly passed on removal into another air."

A singular instance of an endemic fever marked by recurrence and recovery, according to removal or renewal of the cause, is communicated to me by Mr. R. W. Watkins, of Towcester.

"In the year 1846, a farmer's wife, aged about sixty, came under my care with remittent fever. The attack commenced about the beginning of January, and terminated in the middle of March. In 1847, a precisely similar attack came on in the early part of October, and continued till the end of the following January. In 1848, the patient was again attacked in October, and this time the disease continued with varying intensity until March. The locality was a village near the top of a hill, with free currents of air in every direction; the soil a stiff clay. No similar case occurred in the village during either of these attacks. The only cause which could be assigned for the recurrence of this attack, in the winter, was that the house drain after passing under-ground for a few yards, opened into the farm-yard in front of the house. During the winter months this yard was occupied by cattle, and the flow of sewage was necessarily obstructed by the accumulation of litter and manure, and by the trampling of the cattle. The opinion was pressed upon the husband by myself, and confirmed by an eminent physician who was in consultation with me during the case. The farmer was, however, an ignorant man of the old school, and he obstinately refused to admit this explanation. 'It always had been so ever since he was a boy, and no harm had ever come of it.' During the third attack, the poor old man died suddenly, and at the end of spring, the farming stock was all sold. The yard was no longer used as a farm-yard. The drain still remained uncovered, but the sewage had free course down the yard, and into the ditch by the public road. It is remarkable that for seven years afterwards the widow did not have a recurrence of fever; but about the end of that period, circumstances (which I need not particularise) required that the yard should again be used as a farm-yard, and it is no less remarkable that in January, 1856, she was again attacked in a similar manner, though not so violently, and the symptoms were alleviated in a month. In the following October the disease returned, presenting the same character and the same intractability that it had done in former years, and in this instance it continued the whole of the winter."

In April of the present year I received a note from Mr. Watkins, in which he informs me that after the last attack, an underground drain had been laid from the house in which the patient lived, through the farm-yard into the public road, and that she had enjoyed very good health during the subsequent winter. Mr. Watkins justly remarks, "My previous suspicions of the origin of the fever have certainly been confirmed by the result."

Diarrhœa, although it may be classified under certain circumstances, and in certain of its varieties under the second and third heads of my division, sometimes belongs exclusively to the first, now under consideration. We have a very pure example of diarrhœa from the presence of a floating poison, in what is called the dissecting-room cholera. In some of my experiments with sewer air, diarrhœa was the leading result, a fact proving indisputably that the introduction of a poison by inhalation may be productive of alvine flux. I have many illustrations of diarrhœa thus locally generated, but one supplied to me by Mr. Milner is least liable to fallacy :—

"When making my monthly inspection," says Mr. Milner, "on the 1st of March, 1848, I found that the basements of two of the four wings of the New Prison (Wakefield) were flooded, but the basements of the other two wings were not flooded. The two wings which were flooded, were B and C, and contained three hundred and ninety prisoners; the wings which were not flooded were A and D, and these contained three hundred and thirty prisoners.

"It was found that the flooding was caused by the overflow of a drain, arising from the drain having become stopped at its outlet by a plank which had been left in it by the workmen when the drain was constructed.

"The drain was freed, and the water got rid of. So long as the ground was covered with water no disease appeared, but as soon as it began to dry, cases of diarrhœa occurred, and during the month forty-three men were placed on the sick list for that disease in B and C wings, while during the month only two cases of diarrhœa occurred among the prisoners in A and D wings. The forty-three cases in B and C wings were placed on the sick list on the following dates."

| Dates. | Cases. | Dates. | Cases. | Dates. | Cases. |
|---|---|---|---|---|---|
| 4th ...... | 1 | 14th ...... | 3 | 24th ...... | 2 |
| 5 ...... | 1 | 15 ...... | 2 | 25 ...... | 1 |
| 8 ...... | 5 | 16 ...... | 4 | 26 ...... | 1 |
| 9 ...... | 6 | 17 ...... | 1 | 27 ...... | 2 |
| 10 ...... | 9 | 20 ...... | 2 | 29 ...... | 1 |
| 13 ...... | 1 | 23 ...... | 1 | | — |
| | | | | | 43 |

In reference to the poison by which diarrhœa is produced from cesspool emanations, I would prefer to speak cautiously. Yet there are some facts which, taken accumulatively, are strongly favourable to the idea that one especial poison may be *the* poison. I allude to sulphuretted hydrogen. I have shown that this gas is a steady constituent of cesspool air, and I found in my inquiries as to the physiological effects of the gas, that when breathed in such small proportions as not to produce death by its immediate effects, vomiting and free and painful diarrhœa were the marked symptoms. The diarrhœa also continued for some time after the animals were removed from the presence of the poison, as though either a period of time were required for its perfect elimination from the body, or, as if by its presence in the body it had excited changes in the excreting alimentary process which would not abate on the mere withdrawal of the cause.

It is extraordinary, too, how small a dose of the poison is sufficient to produce prominent symptoms. An atmosphere charged only with 0·056 of the gas, is competent, if breathed for an hour or two, to produce tremors, rapid respiration, and active diarrhœa; the diarrhœa remaining for some hours after the animal has again the luxury of unempoisoned air.

While then I would not dogmatically say that sulphuretted hydrogen is *the* purgative poison from the cesspool, I know it to be a purgative poison from that source, and an active one, too.

The second class of epidemic disorders in the threefold division includes the diseases small-pox, scarlet-fever, communicable typhus and typhoid fever, measles, erysipelas, cholera, and, most strikingly perhaps of all, puerperal fever. These are the diseases of the class common to this country; but yellow-fever and plague belong to the same group.

In collecting illustrations of the spread of these epidemics certain difficulties arise to which I would ask special attention. While the diseases clearly pass from one person to another by direct communication, it seems to me, from observations as to the origin of the first cases, that certain of them may arise without the intervention of any previous case of the same character.

This assertion borders on the supposition of a spontaneous development of a contagious disease. I would not, however, have it implied that I believe in direct spontaneity; but I can have no doubt that in certain instances a contagious disorder may be derived indirectly, that is to say, from a disease of another kind not itself the result of contagion.

One striking illustration which occurred in my own practice will explain well what is conveyed in this argument. In the year 1850, a young married woman at the village of Cople, suffered from peritonitis with perforation of the intestine, a form of disease which I have met with four times in my locality. The case was rapidly fatal, and my assistant made a *post mortem* examination. The operation was done neatly and cleanly. The young man returned home and resumed his duties. The next day he attended a woman in labour. He wore the same clothes as on the preceding day. The labour was natural, and took place at the distance of seven miles from the case of peritonitis. On the third day after labour, the woman was seized with puerperal peritonitis and died rapidly.

Two days after attending the previous labour, and before the puerperal symptoms had presented themselves in that case, the same gentleman attended a second case at a place five miles off in another direction. He wore the same clothes as before. On the third or fourth day this patient was also seized with puerperal peritonitis, and in spite of every treatment, died. Her labour had been quite natural.

The mother of the last named patient, a feeble woman of spare habit, nursed her daughter after her confinement, and washed the linen after her decease. I failed to ascertain if she had previously wounded her hand or fingers, or if she received any prick or scratch in those parts in the act of washing. She was not aware that she had received the slightest puncture or scratch. Within two days after washing the linen, the absorbents of the right arm became inflamed, followed by enlargement and inflammation of the axillary glands. Time was not allowed for suppuration, for the vital powers became rapidly depressed, and she sank within two days in spite of remedial measures and the free administration of stimulants.

The recurrence of these disastrous cases induced me to suspend for a time the obstetric duties of the assistant referred to. This decision was the result of a conviction that he had been, though innocently, the medium of communicating the disease directly in the two first cases, and indirectly in the last case related. This gentleman had been in the habit of keeping his finger-nails very short, and within three days after the occurrence of the last case, the forefinger of his right hand became inflamed under the nail, and very painful. The inflammation extended along the hand and absorbents of the arm to the axillary glands, which were much enlarged. The constitutional disturbance was great, and he was confined to his bed for a week, and unable to go out for more than a fortnight from the commencement of the attack.

Here, then, was an instance of a disease clearly specific in its nature and contagious, originating from a disease not specific and not contagious in the ordinary acceptation of the word.

But this is not the only instance of a disease having an occasional origin in a way that approaches spontaneity. Erysipelas, of the apparently spontaneous nature of which I have several illustrations, may originate as an epidemic without the immediate intervention of any preexisting case.

An illustration of this kind is supplied me by my friend Dr. Richardson, and was as follows: "In the last month of the year 1847, three men residing in an agricultural neighbourhood were engaged threshing corn in a barn. They went to work in the most perfect health, and no epidemic of any kind was prevalent at the time. Erysipelas had not been known in the village for many years. The men approached the end of their task, and were turning up the last bundles of corn. At one time they all three felt a peculiar sensation, which they called a 'sickening sensation.' They thought nothing of it at the moment, but in the course of an hour they agreed that they were all too unwell to continue at their work. They felt cold, shivering, and sickness. They sent for their employer, a retired medical practitioner, who, struck by their symptoms, recommended them to go home and to bed. I visited these men in the evening of the same day, and found them suffering with all the symptoms of a threatened febrile attack. I prescribed for them a saline. On visiting them on the following day, they were all found suffering from erysipelas of the head and face. They were located in different houses, and had no communication with each other after their first separation. In one, the attack was very slight, and the recovery took place in two or three days, the erysipelas confining itself to one cheek and ear. The second case was very severe, but recôvered. The third, which occurred in the oldest of the three (the man being upwards of 60), was of such severity, that the patient was laid up for many weeks, and his life was at one time despaired of. The erysipelas extended over the whole of the right side of the head, pus formed beneath the scalp, and free incisions had to be made. Recovery took place ultimately. These men were located in small cottages, thickly inhabited; but under a strict hygiene the extension of the disease was prevented."

In some instances typhus would seem to have an origin in a local cause, and afterwards to assume the communicable type, its poison being, under such circumstances, reproducible in the affected body. Mr. Rumsey, of Cheltenham, has been kind enough to supply me with a good illustration of this kind of propagation. I will give the narrative in his own words:—

"I recollect, and have good reason to do so, a singular invasion of typhus in my own family. My wife, four children and nurse, were at the sea-side in 1845, at Bude, on the Cornish coast, a fine open sea coast. But a little stream opened into the sea in the midst of this village, and this stream was used as the main sewer of the place. The summer was hot, and the bed of the little river partly dry; and there was at hand some decomposing sea-weed. Sir T. Acland's cottage was close by. Fever broke out in that house. Two of his household perished, others were ill. Three other families in the place were attacked, and two or three cases ended fatally. They carried the fever with them to their respective homes; mine did so, and though the children were spared, our nurse died, after communicating the fever to another servant in Gloucester, who also died. I caught the fever in a milder degree, and soon recovered. Several villagers at Bude were attacked."

Another illustration, not less interesting, is by my friend Dr. Spencer Thomson, of Burton-on-Trent:—

"The following facts," writes Dr. Thomson, "respecting the incubation of typhus fever, may be valuable from the peculiar circumstances of the case, and from my being able to state every particular with well-marked exactness. Ten years ago, I took my wife and eldest child to Edinburgh on a visit. With us there went as nurse a country girl from this neighbourhood. During our stay in Edinburgh, and only two or three days before we left, the nurse received permission to go into town with one of my mother's servants, an Edinburgh girl. They walked about a good deal, got fatigued, it being the month of June, and went to have tea with the Edinburgh girl's friends, who lived in one of the old town wynds or closes. After our return to England, the girl never seemed well, complained of constant dull headache, had a dusky look about the skin, and was in a state of depression both mental and physical. It was, however, within a day or two of completing the six weeks from the date of our leaving Edinburgh before actual feverish symptoms compelling retirement to bed were developed. The girl had a most severe and dangerous attack of typhus fever, which lasted from the time of her taking to bed till her convalescence, full thirteen weeks, that is to say, till she was well enough to be removed from my house. I had been accustomed to see much of the Edinburgh fever during my studentship. I had been away from Edinburgh for ten years, and have often remarked that I had not in the ten years seen a case of the old Edinburgh typhus fever till it occurred in my own house in the person of this nurse girl, who brought it, I have not the slightest doubt, from Edinburgh with her. She stated afterwards, that when she went into the house in the old town where she took tea, she perceived a sickening smell which she never got rid of. She felt ill ever afterwards. I sent my wife and child from home, and kept the case isolated, and thus prevented the disease from extending in my own house; but the girl's mother, who nursed her, had the disease in a much milder form, and one of her younger children, who took it from her, had it milder still. It seemed as if the virulence of the fever was gradually subdued under the influence of a pure country air."

While typhus, erysipelas and puerperal fever would thus seem to have the power of originating in the absence of direct contagion, and, having so originated, of becoming themselves communicable, there are, I think, no proofs of the propagation of any other of the diseases included in the present division except by direct transmission. Scarlet fever, it is true, breaks out often in a manner so sudden and mysterious, that I wonder not at the idea of its spontaneous development having been believed in by some of the older observers. Yet I believe that, on inquiry, every case, however insidious in its approach, has a previous case for its base. Such is the concurrent testimony of all who have favoured me with replies to my inquiries.

But the insidious nature of the scarlet fever outbreak, the way in which the poison hides itself, if I may be allowed the expression, this is the marvel. I am indebted to Dr. Richardson for two illustrations of the insidious propagation of this disorder. I shall quote one of them in full :—

"In a village in Essex" (I am reading from my informant's narrative), "the poor of which were partially under my care in the years 1847 and 1848, there was a severe outbreak of scarlet fever, which secured many victims amongst the younger population. A man residing at the extremity of the village, and on an eminence, had a child seized with the disorder. He had three other children, the whole of whom he at once sent away to their grandmother, who was residing at another village eight miles off. The child attacked with the disease suffered from it in its malignant form and died. This was the last case that occurred at that time in the village. On my suggestion, none of the other children were recalled for two months, and the house was in the meantime whitewashed, and, together with the bed linen and every other material that could be considered capable of retaining the disease, was thoroughly cleansed. At the end of the two months, one boy returned home; he had not been in the house a day before he was seized with the premonitory symptoms of the disorder. He had the disease in its severest form, and in spite of all treatment, died. A period of between four and five months elapsed, in which interval the same cleansing processes were carried on as before; and the parents, anxious to have their children back again, and thinking that all danger must now necessarily be passed, recalled their last remaining boy to them. There had been no case in the village during the time intervening; but this boy, on returning home, was seized exactly in the same manner as the previous one, and also died. What became of the last child of these unfortunate people I do not know, as I left the neighbourhood soon afterwards."

The following illustration from Mr. Augustin Prichard, of Bristol, is a curious example of transmission of scarlet fever through clothes. It occurred in his own family :—

"My eldest boy," he states, "eight years old, went on a visit to a distant part of the country and caught scarlet fever, a man servant in the house which he visited having had it before, and there being a few scattered cases in the neighbouring village. I fetched the boy home after the eruption disappeared, but would not allow him to communicate with his brothers and sisters for two months afterwards. About that time, a servant girl in my house was set to work to dust and brush a great coat, in which my son had been enveloped during his journey home. A day or two afterwards she had a severe sore-throat, which we attributed to cold. One of the children slept in the same bed on the 2nd of May while she was ill, but was separated from her after that night. On the 12th, he had fever, followed by the eruption of scarlet fever. Two other of my children were soon seized in a similar manner, but all got well. This was evidently a clear case of communication through the clothes, as there was not another case in the neighbourhood, and had not been for a very long time."

I have the authentic record from Dr. Richardson of an unpublished case where scarlet fever was undoubtedly conveyed by a letter, and led to disastrous results: "A gentleman well known in the scientific world had some friends living some miles away, whose family were suffering from scarlet fever. The gentleman had given strict orders that all his letters should be brought to himself personally. One morning, however, his child transgressed the rule and carried a letter to its mamma, who read it and sent it down by the child to the husband. The letter was from a member of the infected family; there was no other kind of communication between the families, and no disease in the neighbourhood. The mother and the child each took scarlet fever simultaneously, and both fell victims to its virulence."

That small-pox is propagated purely by contagion is a fact which need not be insisted on; and I should not stop to dwell on this fact, but that I have at hand an illustration showing that the alvine excreta of the small-pox patient may convey the disorder:—

In the autumn of 1847, in a county town, a young man, the son of a miller, returned home from a village many miles off in consequence of illness and inability to work. His father lived at a mill which is situated on a high eminence about a sixth of a mile from the town. About a hundred yards from the mill, and running down the descent, was a double row of cottages, neatly built, and inhabited by clean and industrious people. The illness of the youth resulted in a case of confluent small-pox, and terminated fatally. From the miller's house there was a surface drain which ran from the mill down by the cottages nearest to those on the left hand side, the rows of cottages being separated by a road at least ten yards wide. The miller's son died rapidly, and his clothes were destroyed, but the excreted matters from his body, with other refuse from the house, were floated down the drain in their way to the cesspool which was at the bottom of the hill. In the course of two or three days, there was scarcely a child in the cottages on the left hand side that was not prostrated with the disease, but few of them having been vaccinated, and several died. One or two cases occurred on the right hand side, but these were very limited in number as compared with the others.

The houses being isolated from the town, the parish authorities were enabled to set up a strict quarantine. At the suggestion of their medical officers, two men from the workhouse were placed on the road between the town and these cottages, and allowed no child, woman or nurse to enter the town; a messenger being appointed to make purchases for them. The result was that the disease was confined almost entirely to this particular spot. One or two cases which did occur, in every case by intercommunication, were also placed under supervision, and the town was entirely saved from the ravages of an epidemic which had once or twice proved most fatal in every part.

I spoke in the first division of my paper of cases of typhus, endemic in character and non-contagious. There might be expected from me in this place many illustrations of what is often called contagious typhus, and of its modes of transmission. But, although there seem at first sight, abundant evidences of the contagious character of both typhus and typhoid fevers, the evidences, when closely analysed, certainly do not, to my mind, convey such proof in favour of transmission of these disorders by direct communication, as occurs in cases of small-pox, scarlet fever, or true Asiatic cholera. I believe in the possibility of the transmission of these fevers, because the details supplied by various authors at various times appear convincing; but this is certain, that in country practice, where the isolation of cases renders the study of propagation most easy, my own observation, and that of all who have aided me in this inquiry, points to the conclusion, that the fever we see in practice, and which sometimes assumes the true typhus type, and sometimes the typhoid, with the bowel complication, is confined to special localities, is dependent on local causes, and is not propagated by an affected person coming into contact with unaffected persons out of the fever locality. In some cases, truly, persons who live apart from the diseased community, may and often do suffer from the malady by going personally into an affected spot to fetch it; but it is not brought to them; and, when they who enter into the endemic circle, and so contract the disorder, return themselves to their own healthy parts, although they may pass through all the phases of the disease they have contracted, they do not, as a general rule, introduce the endemic disorder into their own healthy community.

It may be argued that persons coming from healthy into fever districts, come often to visit afflicted friends, and in the course of the personal visit contract the disease. This is possible but doubtful; for if a person could thus contract fever, he could communicate it in the same way on return to his own district. Further, I have seen the disease excited in a patient from entering an affected locality, without his having been in contact at all with any of the patients of the district. The following illustration is in point :—

A gentleman had often complained of the offensive smell in the lower part of his house on going down in the morning ; and was compelled to open the windows and doors for some time every morning, in order to remove it. This arose from a neighbouring drain, and the emanations have frequently been so powerful as to have tarnished the candlesticks in the kitchen, and the brass handles of the doors, not only of his own house but of the adjoining house. In August, 1857, this gentleman was seized with fever, which he had so severely as to be confined to his bed for four weeks. During his illness, a nephew from another part of the country, visited next door, but had no intercourse whatever with his uncle. The nephew returned home, and was directly afterwards seized with fever. After the gentleman's recovery, a shop-girl was also seized with fever, which went through precisely the same course.

I have a great variety of illustrations of the reception of disease in this way.

I cannot too strongly repeat, that in cases of fever arising clearly from local causes, the disease does not seem to spread out of the circle of those causes. If, therefore, there is a form of fever, which is communicable by personal intercourse, I see nothing for it, but to assume that such fever, however analogous in symptom to our country endemic fever, is different in origin; that it is not lighted up in the first instance from the emanations of decomposing organic matter, but, like scarlet fever and small-pox, by a poison which is reproducible only in the living organism.

In the cases of fever which have been supplied to me, it is peculiar how few could be traced to such method of transmission.

The following illustration, from the pen of Mr. Eddowes, of Pontesbury, is the best of the series. His narrative runs as follows:—"I once attended some cases of typhus, which seemed to have been transmitted clearly by contagion. The first case occurred at the house of a carrier, who attended a neighbouring market once a week, and had to pass through a village where typhus was present. This man was taken ill with decided fever, on September 24th. From his house the disease was carried by a woman who waited on him to a hill-side at a considerable distance. Here it spread. The habitations on the hill were open on all sides, but about twenty suffered from the disorder; and the locality remained infected for full three months, and one patient, a boy, died. In one miserable small house, built of turf, dark and damp, having only two small windows, and but one sleeping apartment on the ground floor, there were seven sufferers."

Respecting the remaining diseases of this class, I shall refer only to cholera, concerning the propagation of which many histories of considerable value have been communicated to me. From the analysis of these, from personal observation, and from the general evidence in the literature of the profession, the evidence to me appears to establish to a demonstration, that Asiatic cholera belongs purely to the second division of the classification before the society. As is common, all the illustrations go to connect cholera, as fever, with uncleanliness; with small and dirty dwellings, pigstyes, and middens, cesspools and foul streams.

But in the midst of all these analogous externals, there stand out in all instances, *the imported case, or the imported poison*, and the visible *spread of the disease* out of the distempered locality to wherever the human feet can carry it.

Without perverting the verse of our great poet I might indeed, by change of a word, express from one of his passages a new fact.

> "Cholera is like a circle on the water,
> Which never ceaseth to enlarge itself,
> Till by wide spreading it is brought to nought."

I will give one illustration, which occurred to myself, of the spread of cholera by transmission of the clothes of a cholera patient.

In 1854, no cases of cholera were known in the county of Bedford, when it broke out in the village of Ridgemount, and eleven cases occurred from first to last, all of which were fatal. On careful inquiry as to its origin, it was clearly ascertained that the first case occurred in a man whose son had died of cholera in London, a week or two before, and whose clothes were sent down to the country. The poor man unpacked the bundle of clothes himself, was seized with the disease, and died. His case was the nucleus of the rest.

As showing to what extent local influences modify the spread of cholera, the following facts from Mr. Milner, medical superintendent of Wakefield Prison, will, I am sure, be received as of great value.

"The prison at Wakefield stands on about eighteen acres of low-lying land, situated on the windward side of the town.

"The soil is coarse clay, and the ground rises a little from the banks of a brook, which skirts a portion of the boundary walls. There are numerous blocks of building within the walls, but the principal ones are those called respectively, the Old Prison, the New Prison, and the Women's Prison. The old prison is a curved block of building, forming the greater part of a circle. It partially surrounds the house formerly occupied by the governor, but is separated from it by a space of about fifteen or twenty yards. It stands on the lowest part of the ground, and at that time (January, 1849) was not provided with any means for providing warmth or ventilation, and as the ground on which the building stood was very inefficiently drained, the cells were damp, cold, and badly aerated.

"The new prison is a K shaped building, consisting of four wings, constructed on the Pentonville plan; it stands upon a hill about sixteen feet above the ground on which the old prison was built. The ground is thoroughly drained; the foundations of the building are laid in concrete, and the building itself is well warmed and ventilated.

"The women's prison is built on the same plan, and possesses the same advantages.

"In January, 1849, we had cholera in the prison. At that time there were about 230 prisoners in the old prison, 400 convicts and 300 West Riding prisoners in the new prison, and 60 women in the women's prison.

"We had 27 cases of cholera in the old prison, and not one either in the new prison, or in the women's prison.

Diarrhœa prevailed in every part of the establishment, but in different degrees; for while in the new prison and in the women's prison, the amount of diarrhœa if distributed equally over the average daily number in confinement, would have give only half a day's sickness per head, in the old prison each prisoner would have had six and a half days sickness, shewing that the essential cause of the disease was in existence, and acting over the whole prison, but that its action was intensified thirteen times by the bad sanitary condition of the old prison."

The diseases of the epidemic series which I would enumerate as belonging to the third division of the classification on the table, are, common epidemic catarrh, a form of diarrhœa which affects the poorly clad population on the event of a sudden fall of the barometer; croup, and I am inclined to add pertussis.

Diseases having an origin in meteorological changes have this peculiarity. They break out all at once over a wide surface of country, and after attacking in the same day and I had almost said at the same hour, great numbers of persons, pass away in the same general manner as they came, leaving only their consequences.

I have now kept records of the diseases of my district with counter records of meteorology for some years, and the data supplied are of interest. But as I must consider the time of the society, I cannot, as I had intended, enter deeper into this question at the present moment.

But before I conclude, there are two or three points relating to the nature of those poisons by which the first and second classes of diseases are produced, to which reference must be made.

Concerning the nature of the poisons themselves, it occurs to me that those producing endemic non-contagious diseases may differ from those which produce the contagious diseases, in this simple particular, that the first-named poisons, however subtle and diffusive, are inorganic, and lose their influence in the body which receives them; while the second are organic, and being capable of reproduction under favouring conditions, are propagated in the animal body, finding, in fact, in the animal body, the conditions *most* favourable for their propagation and increase.

These organic poisons, eliminated by the sick man, and finding no favourable seed ground in another person susceptible to them, may lie in temporary death and disuse for a season. But once set at liberty and diffused by air or water so as to approach the susceptible individual, they put forth a new existence, and an epidemic starting from one centre is the result.

Another point on which I would dwell in regard to these poisons, whether of the first or second series, and whether organic or inorganic, is,—that given a medium for their transmission into the body, it is of little moment how they are introduced.

There has been much dispute lately as to air and water, as the mediums of special diseases. The end of the dispute lies, I believe, in accepting both as possible mediums, and in looking on the occurrence of one or the other as the medium, as a mere matter of accident. I have many illustrations in hand in which the endemic typhus of which I have spoken so much, was conveyed by water. I have many illustrations which, without the possibility of a doubt, prove that the same disease was conveyed by air. I cannot refrain from giving one example bearing on this last point.

The narrative reads like one of my own experiments described in the opening of the paper, except that the animals were men, the box a prison, and that the cesspool air fed the box by the plenum in lieu of the vacuum principle. The history is given me by Mr. Milner of Wakefield, to whom I have already proffered my deep obligations. Mr. Milner writes as follows :—

"In the early part of 1849, I visited a number of prisons for the purpose of selecting convicts to be removed to Wakefield. Among the prisons so visited was Lincoln Castle. I there found thirteen men under sentence of transportation, twelve of them were then either suffering from fever, or convalescent from attacks of fever. On inquiry it appeared that a block of cells constructed on the Pentonville plan had been built, and prisoners had been placed in them, I think for the first time, in October, 1848. By the end of the year almost every prisoner who had been placed in these cells was ill, and the greater part had continued fever; at the time of my visit on the 5th of June, 1849, the prisoners had all been removed from these cells.

"I examined the cells, and found them well built, dry, and provided with good arrangements for warmth and ventilation; they were also perfectly clean, and evidently had been kept so when occupied. They were all provided with water-closets which were clean and in good order. I then inquired about the drains, and the mystery was at once solved. There were no drains from the prison, but all the sewage from these cells was conducted into a closed cesspool, which I was most carefully assured had been made *quite air-tight*. The result of this arrangement was that when the tension of the disengaged gases in this air-tight cesspool became greater than the water sent into the syphon trap of the water-closets, large quantities of these gases escaped into the cells, as one of the prisoners told me, 'with a noise like thunder, and a stench that would poison the devil.'

"I reported to the Home Secretary that I had declined to remove any convicts from Lincoln to Wakefield, and gave the prevalence of fever in the former gaol as my reason for so doing; and on my report, Mr. Perry, one of the Prison Inspectors, was sent down to Lincoln."

The last topic on which I shall touch relates to the influence of season, temperature, moisture and other modifications of the atmosphere in their influences on the spread of those diseases which have their origin in an organic and reproducible poison. Much has been written and said on this point, and, as is common, extremes of view have been taken. Meteorologists have denied contagion; contagionists have ignored meteorology; but whoever will remove from the combatants and look calmly on will see that both have a kernel in their cracked nut. Given certain meteorological conditions in tropical India, and the vaccine virus, and even small-pox virus, loses its power. Given other meteorological conditions, and the virus has an activity which is unapproachable. Now, who in this matter is right or wrong? The palsied contagionist with the virus on his lancet point which won't go, or the triumphant meteorologist with his eye immoveable on the thermometer or the rain-gauge? Looking on without bias, I see them both correct in their special ways. I see, in short, that the virus is necessary to the production of the disease, but that the virus can only act during conditions of heat or cold, dryness or moisture, favourable to its development.

I would suggest that this same relationship between the poisons of spreading diseases and atmospheric conditions is universally sustained, and with this suggestion would respectfully submit the whole argument to the more matured learning of the Fellows of this Society.*

---

* In communicating this paper to the *Transactions* of the Epidemiological Society, I would add that, besides those of my professional brethren mentioned therein as having contributed facts and cases relating to the subject under discussion, I am in like manner deeply indebted to the following gentlemen for interesting communications, several of which have been available in one chapter of the "Essay on Malaria". I beg thus publicly to acknowledge the kind assistance of—Mr. H. W. Bailey; Dr. Fred. Brown; Mr. W. I. Cox; Mr. Hy. Davis; Dr. John Davy; Dr. James Heygate; Mr. J. A. Hingeston; Mr. W. H. Hole; Mr. J. H. Houghton; Mr. E. L. Hussey; Mr. Wotton Isaacson; Mr. W. C. Lake; Dr. D. Lietch; Dr. Lauder Lindsay; Mr. Septimus Lowe; Dr. Mackinder; Mr. G. May; Dr. McIntyre; Dr. Moffat; Mr. J. G. Moyle; Dr. Pickells; Mr. Geo. Rigden; Dr. G. W. Spence; Mr. T. Spurgin; Mr. E. C. Summers; Dr. Thos. Thomson; Mr. Geo. Todd; Mr. Wm. Walker; and Dr. R. U. West.

